# 肺腺癌和鳞癌中VEGF-C和新生淋巴管表达的预后价值

**DOI:** 10.3779/j.issn.1009-3419.2011.10.02

**Published:** 2011-10-20

**Authors:** 学利 代, 文利 王, 阳 申屠, 杰 张

**Affiliations:** 1 200030 上海，上海交通大学附属胸科医院/上海市肺部肿瘤临床医学中心胸外科 Department of Thoracic Surgery, Shanghai Chest Hospital/Shanghai Lung Tumor Clinical Medical Center, Shanghai 200030, China; 2 200030 上海，上海交通大学附属胸科医院/上海市肺部肿瘤临床医学中心病理科 Department of Pathology, Shanghai Chest Hospital/Shanghai Lung Tumor Clinical Medical Center, Shanghai 200030, China

**Keywords:** 肺肿瘤, 血管内皮生长因子-C, 新生淋巴管, 预后, Lung neoplasms, Vascular endothelial growth factor-C, Lymphangiogenesis, Prognosis

## Abstract

**背景与目的:**

血管内皮生长因子-C（vascular endothelial growth factor-C, VEGF-C）是VEGF家族的成员之一，且已被证明是相对特异的血管内皮生成因子，其与受体VEGFR-3结合后可激活淋巴管的生成，为肿瘤的淋巴结转移创造有利的条件。本研究旨在探讨VEGF-C和新生淋巴管在肺腺癌和鳞癌中的表达特点及其预后意义。

**方法:**

以跨粘膜样受体蛋白（podoplanin）标记新生淋巴管内皮细胞，采用免疫组化方法检测98例Ⅲa（N2）期肺腺癌和鳞癌中VEGF-C和新生淋巴管的表达。

**结果:**

VEGF-C的表达率与微淋巴管密度（lymphatic microvessel density, LMVD）之间存在正相关（*r*=0.783, *P* < 0.01）。VEGF-C高表达组的LMVD大于低表达组（*P* < 0.01）。肺腺癌VEGF-C和新生淋巴管表达率明显高于肺鳞癌（*P* < 0.01）。VEGF-C阳性表达患者的生存率明显低于阴性患者（*P* < 0.05），VEGF-C是影响预后的独立因素。

**结论:**

Ⅲa（N2）期肺腺癌新生淋巴管的表达较鳞癌明显。VEGF-C是影响Ⅲa（N2）期肺腺癌和鳞癌患者预后的独立因素。

## Abstract (en)

**Background and objective:**

Vascular endothelial growth factor-C (VEGF-C), a member of the VEGF family, has been proven to be a relatively special VEGF. When binding to its receptor VEGFR-3, it activates lymphangiogenesis, which likely induces lymph node metastasis in tumors. The aim of this study is to characterize the expression and prognostic value of VEGF-C and lymphangiogenesis in lung adenocarcinoma and squamous cell carcinoma.

**Methods:**

Through immunohistochemistry, the lymph vessel endothelial cells undergoing lymphangiogenesis were stained with podoplanin to detect the expression of VEGF-C and lymphangiogenesis in 98 cases with stage Ⅲa (N2) lung adenocarcinoma and squamous cell carcinoma.

**Results:**

The expression rate of the VEGF-C was positively correlated with the lymphatic microvessel density (LMVD; *r*=0.783, *P* < 0.01). The LMVD was remarkably higher in VEGF-C positive expression group than that in the VEGF-C negative expression group. The expression rate of VEGF-C and lymphangiogenesis in lung adenocarcinoma was significantly higher than that in lung squamous cell carcinoma (*P* < 0.01). *Kaplan*-*Meier* analysis showed that the survival rates in patients with positive VEGF-C expression were significantly lower than those in patients with negative VEGF-C expression (*P* < 0.05).

**Conclusion:**

Lymphangiogenesis rates are significantly higher in lung adenocarcinoma than that in squamous cell carcinoma. VEGF-C expression is an independent prognostic factor of stage Ⅲa (N2) lung adenocarcinoma and squamous cell carcinoma.

淋巴转移是肺癌手术后最常见的扩散途径。近年来，肺癌VEGF-C和新生淋巴管的表达与淋巴转移之间的关系引起研究者的广泛兴趣。其中，不同病理亚型肺癌VEGF-C和新生淋巴管的表达的结果尚存争议^[1-3]^。本文通过研究肺腺癌和鳞癌中VEGF-C和新生淋巴管表达的特征及其与患者术后生存率的相关性，以期为临床提供有益的肺癌预后指标。

## 1 资料与方法

### 1.1 患者资料

#### 1.1.1 纳入标准

上海市胸科医院胸外科1999年1月-2003年12月共行肺癌手术2, 850例，入组标准：①达到肺癌完全性切除标准；②术后病理证实为Ⅲa（N2）期非小细胞肺癌（non-small cell lung cancer, NSCLC）；③肺腺癌或鳞癌；④有完整的术后5年随访及生存资料。术后病理诊断不属NSCLC或未能完全性切除者予以剔除。共计入组98例患者，其中肺鳞癌39例，肺腺癌59例。

#### 1.1.2 资料收集

住院资料以上海市胸科医院病案室存档病史资料为准。生存随访资料均来源于上海市疾病预防控制中心。所有患者均进行肿瘤传报。通过SPSS 13.0数据库采集以下数据：住院号、性别、年龄、诊断时年龄、手术日期、手术类型、病理类型、T分期、N分期、分化程度、清扫淋巴结分期组（数）、阳性淋巴结组，术后化疗方案及周期。病理诊断根据2004年世界卫生组织颁布的肺及胸膜肿瘤组织学分类（第4版）标准^[4]^。病理分期按2009版国际抗癌联盟肺癌分期标准进行。

### 1.2 研究方法

统计分析入组患者的临床特征及生存率，以免疫组织化学染色法检测所有病灶VEGF-C和新生淋巴管的表达。比较肺腺癌和鳞癌病灶VEGF-C/新生淋巴管的表达特点，评价病灶VEGF-C和新生淋巴管表达的预后价值。

### 1.3 免疫组织化学研究

#### 1.3.1 研究对象

收集上述98例肺癌患者术后病灶石蜡标本，共计98块。

#### 1.3.2 方法及程序

采取柠檬酸盐缓冲液微波抗原修复法。每例标本切片×2，脱蜡至水。23%H_2_O_2_处理10 min，放入盛有柠檬酸盐缓冲液（pH6.0）的容器中，置微波炉内加热使容器内液体温度保持在92 ℃-98 ℃之间并持续10 min-15 min取出容器，室温冷却20 min-30 min，切片先用蒸馏水冲洗两次，再用PBS冲洗，10%正常山羊血清封闭，室温孵育10 min。VEGF-C和podoplanin一抗均为美国R&D公司进口分装产品，VEGF-C为浓缩液，podoplanin为冻干粉，均用抗体稀释液（福州迈新公司产品）稀释，VEGF-C稀释比为1:50配置成工作液浓度，podoplanin按说明书加入0.2 mL抗体稀释液稀释为浓缩液，再按1:100稀释比配置成工作液浓度。切片以一抗37 ℃温育1 h，PBS冲洗3次。二抗为DAKO公司产品，加兔Envinsion室温30 min温育，PBS冲洗3次。滴加新鲜配制DAB显色液（DAKO公司产品），显微镜下观察5 min-10 min，在显色最佳时用PBS冲洗，中止显色。苏木素复染细胞核，然后水洗、蓝化、脱水、中性树胶封片。

#### 1.3.3 VEGF-C表达的观察和计量

应用日本产Olympus BX51光电显微镜对切片进行观察。VEGF-C在癌细胞内的表达显示胞浆或细胞膜上有黄色或棕黄色颗粒。先在×40倍下观察整体着色情况，继而在×100倍下确定染色区域，再在×200倍下应用Leica Qwin320 plus图像处理与分析系统进行图像检测。病灶VEGF-C表达阳性细胞数所占百分比计分方法：无阳性细胞数为0分；阳性细胞数 < 10%为1分；阳性细胞数10%-50%为2分；阳性细胞数>50%为3分。病灶VEGF-C表达阳性细胞染色强度计分：无色为0分；淡黄色为1分；棕黄色为2分；棕褐色为3分。以上述百分比计分与染色强度计分的乘积获得VEGF-C表达的等级：0分-2分为（-），3分-4分为（+），5分-7分为（++），8分-9分为（+++）。统计每一级别例数，以等级资料进行统计。

#### 1.3.4 新生淋巴管表达的观察和计量

应用日本产Olympus BX51光电显微镜进行观察。先在×40倍下观察整体着色情况，在×100倍下确定“热点”区，即淋巴管密度最高处，在×200倍下进一步区分着色情况，剔除因成纤维细胞染色而选取的细胞间质。新生淋巴管的确定条件为：podoplanin染色为黄色扁平样、单层管壁无肌细胞和周细胞、管腔内无红细胞出现。单个内皮细胞着色亦作为一个计数单位，不以是否形成管腔和管腔内有无淋巴细胞作为计数标准。然后在×400倍视野下选择5个不重复的视野计数，计算每例切片单位视野平均新生淋巴管数，以微淋巴管密度值表示（lymph microvessel density, LMVD），单位为个。以Mean±SD表示。

### 1.4 统计学方法

所有数据的统计分析均采用SPSS 13.0统计软件包进行处理。定量资料的分析采用*t*检验，率或构成比分析采用*χ*^2^检验。生存分析采用*Kaplan*-*Meier*乘积法和*Log*-*rank*检验，相关性分析采用*Pearson*或*Spearman*相关分析。*P* < 0.05为有差异统计学意义。

## 2 结果

### 2.1 肺腺癌和鳞癌中VEGF-C的表达及其与病理因素的关系

肺腺癌和鳞癌中VEGF-C阳性表达呈现棕黄色颗粒（图 1）。98例患者中，共计58例（59.2%）的VEGF-C呈阳性表达。其中腺癌组表达阳性率（71.2%）明显高于鳞癌组（41.0%）（*P*=0.003）（表 1）。VEGF-C的表达与病理类型相关，而与T分期、肿瘤大小、分化程度无关（表 2）。

**1:**
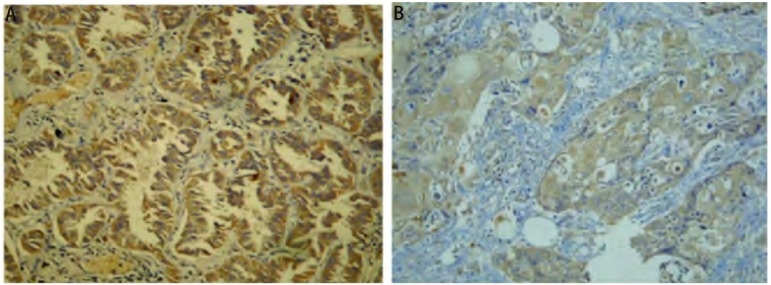
免疫组化法检测肺腺癌（A）和鳞癌（B）中血管内皮生长因子-C（VEGF-C）的阳性表达（×100） The positive expression of vascular endothelial growth factor-C (VEGF-C) in lung adenocarcinoma (A) and squamous cell carcinoma (B) by immunohistochemistry (×100)

**1:** 肺腺癌（A）和鳞癌（B）中VEGF-C的表达 The expression of VEGF-C in lung adenocarcinoma and squamous cell carcinoma

VEGF-C expression level	(-)	(+)	(++)	(+++)
Lung adenocarcinoma	17 (28.8%)	14 (23.7%)	15 (25.5%)	13 (22.0%)
Lung squamous cell carcinoma	22 (56.4%)	1 (2.6%)	6 (15.4%)	10 (25.6%)

**2:** 肺腺癌和鳞癌中VEGF-C和新生淋巴管的表达与病理因素的关系 The relationship between VEGF-C/lymphangiogenesis expression and pathologic factors

Pathologic factors	*n*	VEGF-C expression	*P*	LMVD	*P*
T stage			0.744		0.213
T1	8	4 (50.0%)		5.00±4.00	
T2	70	43 (61.4%)		5.01±3.93	
T3	20	11 (55.0%)		6.05±4.42	
Tumor size (cm)			0.521		0.198
< 7 cm	88	51 (58.0%)		4.82±3.95	
≥7 cm	10	7 (70.0%)		6.60±5.46	
Pathological type			0.003		0.002
Adenocarcinoma	59	42 (71.2%)		6.20±3.81	
Squamous cell carcinoma	39	16 (41.0%)		3.95±3.18	
Differentiation			0.761		0.447
High	11	7 (63.6%)		4.47±4.04	
Middle	49	26 (53.1%)		4.73±3.90	
Low	38	25 (65.8%)		5.74±4.18	
LMVD: lymphatic microvessel density.					

### 2.2 肺腺癌和鳞癌中新生淋巴管的表达及其与病理因素的关系

新生淋巴管非均匀分布于肿瘤组织，大部分出现在肿瘤边缘，而肿瘤内的淋巴管大多塌陷（图 2）。新生淋巴管表达数量以微淋巴管密度值LMVD表示，98例患者中LMVD为（5.00±4.13）个，其中腺癌（6.20±3.81）个，鳞癌（3.95±3.18）个。VEGF-C的表达与病理类型有关，而与T分期、肿瘤大小、分化程度无关（表 2）。

**2:**
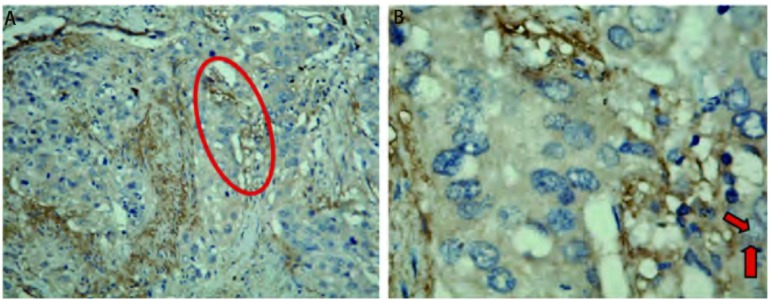
免疫组化法检测肺腺癌和鳞癌中新生淋巴管的表达。A：红色椭圆形区域为新生淋巴管表达区域（×100）；B：红色箭头所指为新生淋巴管（×200） The expression of lymphangiogenesis in lung adenocarcinoma and squamous cell carcinoma by immunohistochemistry. A: The red elliptical area showed the expression of lymphangiogenesis (×100); B: The red arrow showed the the expression of lymphangiogenesis (×200)

### 2.3 肺腺癌和鳞癌中VEGF-C表达与新生淋巴管的关系

98例病灶中，VEGF-C阳性表达为58例，LMVD为（7.67±3.10）个；VEGF-C阴性表达为40例，LMVD为（1.52±1.13）个。VEGF-C表达阳性和阴性者的LMVD间存在统计学差异（*t*=12.35, *P* < 0.01）。98例病灶中VEGF-C的表达与LMVD相关（*r*=0.749, *P* < 0.01）。

### 2.4 生存分析

98例患者的总体5年生存率为38.8%（38/98），中位生存期为（37.53±4.05）个月，肺鳞癌组5年生存率为46.2%（18/39），腺癌组为33.9%（20/59），两组间无统计学差异（*P*=0.273）。

#### 2.4.1 单因素分析

VEGF-C表达阳性患者58例，阴性40例，两者的中位生存期分别为（21.53±2.98）个月和（55.3±4.05）个月，VEGF-C表达阳性者预后较差（*χ*^2^=35.48, *P*=0.001）（图 3A）。如将患者分为高LMVD组（≥5个）和低LMVD组（< 5个），则两者的中位生存期分别为（21.23±2.22）个月和（53.74±3.24）个月，高LMVD者预后较差（*χ*^2^=35.48, *P*=0.001）（图 3B）。

**3:**
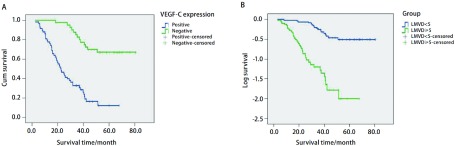
*Kaplan*-*Meier*累计生存时间曲线分析。A：VEGF-C阳性表达患者和阴性表达患者的*Kaplan*-*Meier*累计生存时间曲线；B：高LMVD组患者和低LMVD组患者的*Kaplan*-*Meier*累计生存时间曲线 *Kaplan*-*Meier* cumulative survival time curves analysis. A: *Kaplan*-*Meier* cumulative survival time curves of VEGF-C positive expression group and VEGF-C negative expression group; B: *Kaplan*-*Meier* cumulative survival time curves of higher LMVD (≥5) and lower LMVD (< 5)

#### 2.4.2 多因素分析

采用*Cox*模型对患者年龄、性别、T分期、肿瘤直径、病理类型、分化程度、VEGF-C表达及LMVD进行多因素分析，发现仅病灶VEGF-C表达是影响患者术后生存的独立预后因素（表 3）。

**3:** 肺腺癌和鳞癌患者生存期的预后因素（*Cox*回归分析） Prognostic factors for survival in patients with lung adenocarcinoma and squamous cell carcinoma (*Cox* regression model)

Characteristics	*P*	Hazard ratio (HR)	95%CI
Age	0.128	1.028	0.992-1.065
Gender	0.461	0.754	0.356-1.598
T stage	0.950	0.983	0.576-1.678
Tumor size	0.130	3.367	1.296-8.747
Differentiation	0.140	0.538	0.328-0.882
Pathological type	0.960	1.973	0.889-4.331
LMVD	0.350	1.177	1.012-1.370
VEGF-C expression	0.001	1.862	1.464-2.386

## 3 讨论

早期肺癌患者手术后远处转移的发生率高达60%，成为术后远期死亡的重要原因，并最终影响总体疗效^[5]^。介导远处转移的主要途径是淋巴和血行，以淋巴转移最为常见。由于淋巴管的基底膜薄且不连续，不能对肿瘤细胞构成有效屏障，因此肿瘤细胞易于侵入淋巴管，此外，新生淋巴管也可从外部长入肿瘤内部，使肿瘤细胞被动进入新生淋巴管内^[4]^。

随着近年来淋巴内皮细胞标志物的逐渐发现，淋巴转移机制的研究有了明显的进展^[6]^。Podoplanin是在肾小球足状突细胞首先证实的一种整合胞质胞膜粘蛋白，一度认为，除皮肤某些血管内皮细胞外，podoplanin只表达于淋巴管内皮，后发现其在Ⅰ型肺泡上皮、乳腺肌上皮及食管、肺、肝、直肠和乳腺来源的癌细胞上亦有表达^[7]^。但相比于曾广泛应用的VEGFR-3和LYVE-1，由于podoplanin在正常肺组织及肺癌病灶的血管内皮上无表达，因而新生淋巴管易于甄别，迄今仍是相对准确的新生淋巴管标记物。

VEGF是一个细胞因子家族，包括VEGF-A、VEGF-B、VEGF-C、VEGF-D、VEGF-E和胎盘生长因子等。VEGF-C的受体有两个：VEGFR-2和VEGFR-3，均属于酪氨酸激酶受体，特异性地存在于内皮细胞。相关研究^[8, 9]^显示，VEGF-C与VEGFR-3结合可发挥明显、特异的促淋巴管生成作用，同一组织中VEGF-C与VEGFR-3的结合力是VEGFR-2的3倍，使得VEGF-C的促淋巴管生成作用成为主导，是促进淋巴管新生最重要的因子。肿瘤组织中过表达的VEGF-C不仅诱导淋巴管的新生，而且使肿瘤组织外周淋巴管直径增加，增加了肿瘤细胞与淋巴管的接触面积，可能籍此增加肿瘤细胞经淋巴系统转移的机会。本研究显示，对Ⅲa（N2）期患者而言，随着VEGF-C表达的增强，新生淋巴管的数量也逐渐增加，且VEGF-C表达者新生淋巴管的数量明显高于无表达者，VEGF-C的表达与新生淋巴管之间存在正相关性，提示肿瘤组织中VEGF-C的表达可能促进新生淋巴管的生成。有研究^[10]^认为VEGF-C促进NSCLC的淋巴管生成，有利于肿瘤细胞进入淋巴管发生转移，尽管存在异议^[11]^，但作者支持前述新生淋巴管的分子机制。

有研究^[12, 13]^报道，肺癌病灶VEGF-C表达与患者淋巴转移密切关联，在发生淋巴结转移的病灶边缘淋巴管密度明显升高，新生淋巴管可作为淋巴结转移的预测因子。在生存分析的研究中，多数提示VEGF-C是影响预后的因素^[12-15]^。此外，有学者^[13]^报道病灶VEGF-C的表达与NSCLC淋巴结转移、淋巴系统受侵呈正相关；而病灶淋巴管密度则与VEGF-C表达、淋巴结转移、临床分期呈正相关，认为VEGF-C的表达在NSCLC的发生、发展过程中起重要作用。本研究的单因素分析中，病灶VEGF-C表达患者与无表达者的中位生存期差距明显，VEGF-C表达者预后较差。多因素分析亦显示病灶VEGF-C表达是影响患者生存的独立因素，与前述报道结论一致，可能与VEGF-C的高表达促进肿瘤细胞经淋巴转移有关，因此VEGF-C的高表达可作为肺癌的高危预测因素。对于病灶新生淋巴管表达，本研究仅单因素分析提示高表达组的中位生存期明显低于低表达组，新生淋巴管数量较多者预后较差，但多因素分析并未显示新生淋巴管密度是独立的预后影响因素。有研究^[11]^认为，肺癌患者本身所具有的淋巴管在肿瘤转移过程中的作用比新生淋巴管更重要，新生淋巴管可能起到了促进肿瘤向正常淋巴系统转移的作用。

研究发现，肺腺癌的微血管密度明显高于肺鳞癌^[16]^，但是有关肺腺、鳞癌新生淋巴管的差异结论不一^[1-3]^。本研究中，腺癌病灶VEGF-C表达率（71.2%）明显高于鳞癌组（41.0%）（*P*=0.003），腺癌病灶新生淋巴管密度平均为（6.20±3.81）个，亦明显高于鳞癌组（3.95±3.18）个（*P*=0.001)，且二者与患者年龄、性别、T分期及肿瘤分化程度无明显相关性。本研究提示肺腺癌VEGF-C的高表达，可能较肺鳞癌更明显促进新生淋巴管的生成，使癌细胞经淋巴转移的机率更高，从而影响患者的术后效果。至于两组之间并无明显生存差异，可能因本研究以Ⅲa期患者为研究对象，患者已然出现纵隔淋巴结转移，提示肺癌晚期（Ⅲa）期患者病理分型并非影响生存率的主要因素，这也从另一个侧面印证了病理分期对预后的评估价值。当然，由于两组病例数存在明显倾斜（腺癌59例*vs*鳞癌39例）且总体病例数有限，有待扩大样本量进一步研究。

肺癌2009新的国际分期将肿瘤直径≥7 cm者划归为T3，预后明显差于肿瘤直径较小者。本研究虽未发现≥7 cm肿瘤VEGF-C和新生淋巴管的明显高表达，可见肿瘤直径的增大并未促进VEGF-C和新生淋巴管的表达，但须提及的是，本研究中肿瘤直径≥7 cm的比例较低（10/98），而其VEGF-C的表达率却明显高于肿瘤直径 < 7 cm者（70% *vs* 58%），而且LMVD亦较高（6.60±5.46 *vs* 4.82±3.95），因此，亦有待进一步扩大研究样本量才能获得更为可信结论。
